# Rare Case of Surgical Treatment of a Giant Aortic Arch False Aneurysm

**DOI:** 10.1055/s-0039-1678553

**Published:** 2019-02-22

**Authors:** Vladimir Shlomin, Yury Didenko, Igor Drozhzhin, Petr Puzdriak, Pavel Bondarenko, Nadezhda Grebenkina

**Affiliations:** 1 Department of Vascular Surgery, City Multiservice Hospital No. 2, Saint Petersburg, Russia

**Keywords:** aortic arch false aneurysm, pseudoaneurysm, temporary bypass

## Abstract

The authors present a clinical case of a 44-year-old male patient with a chronic giant aortic arch pseudoaneurysm with a diameter of 136 × 72 mm. The open resection of false aneurysm was accomplished without artificial circulation. Repair was performed with temporary ascending-to-descending and brachiocephalic bypass without cardiopulmonary bypass.

## Introduction

Damage to the aorta as result of blunt chest trauma is a rare and dangerous condition with a high mortality rate (80–90%) due to massive bleeding. However, in some patients, despite the rupture of the thoracic aortic wall, mediastinal tissues “restrain” the hematoma, preventing the development of fatal bleeding. Subsequently, this leads to the formation of a false aneurysm. According to Parmley et al, 1 the most frequent site of aortic rupture in such injuries is the zone of its isthmus. This location of injury at the isthmus has been attributed to embryogenesis of the aorta and anatomical features. 1 The localization of the rupture in the region of the aortic arch, as in our case, is an extremely rare observation.

## Case Presentation

A 44-year-old male patient was admitted to our Vascular Surgery Department.

In 2001, the patient was in a traffic accident, resulting in a blunt injury to the chest and pelvis. This, presumably, was the mechanism of development of an aneurysm of the aortic arch.

In 2012, on the plane X-ray of the chest, an abnormal mass lesion was found, but computed tomographic (CT) verification was not performed due to unknown reason.

**Fig. 1:**
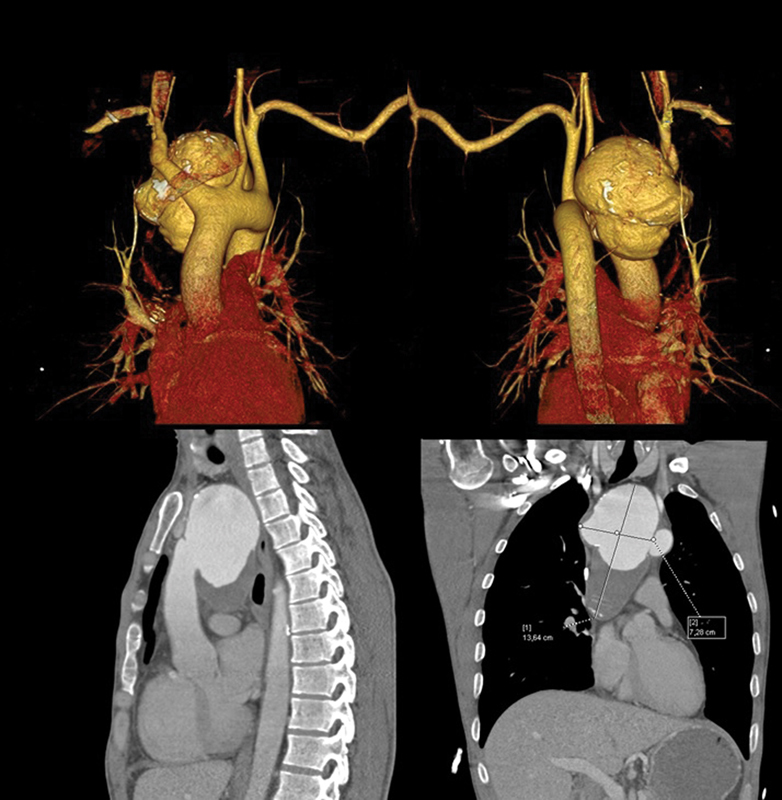
Preoperative CT aortography of pseudoaneurysm of the aortic arch in the posterior mediastinum.

In 2014, the patient was hospitalized in our department when we confirmed the diagnosis of the aortic arch pseudoaneurysm ( Fig. 1 ). CT imaging identified a giant pseudoaneurysm with maximum size 136 × 72 mm. The size of posterior aortic arch wall defect was 28 mm. There were no signs of aortic dissection.

We performed an operation—the elimination of the aortic arch pseudoaneurysm and posterior wall tear and false aneurysm in the mediastinum without the use of cardiopulmonary bypass.

The position of the patient was on hs back with his left hand fixed above the head.

**Fig. 2:**
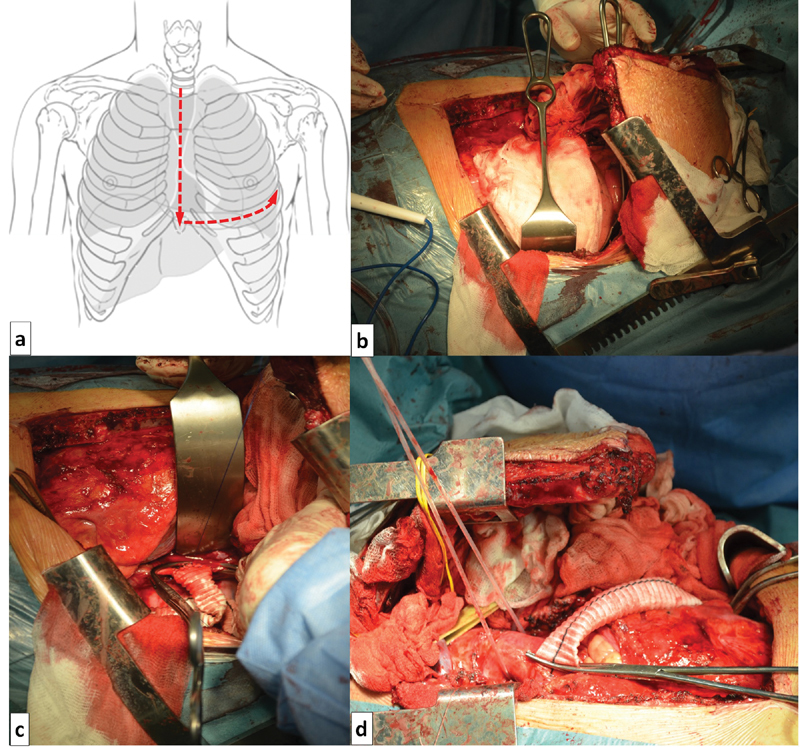
L-shaped median sternotomy with a left thoracostomy in the fifth intercostal space: ( **a** ) schematic, ( **b** ) intraoperative picture of the approach, ( **c** ) distal anastomosis between temporary shunt and descending thoracic aorta, ( **d** ) proximal anastomosis between temporary shunt and descending thoracic aorta.

Under total anesthesia, through the L-shaped median sternotomy and left 5th intercostal thoracotomy, we identified and extracted the ascending aorta, aortic arch, left common carotid and subclavian arteries and mid part of descending aorta ( Fig. 2 ).

**Fig. 3:**
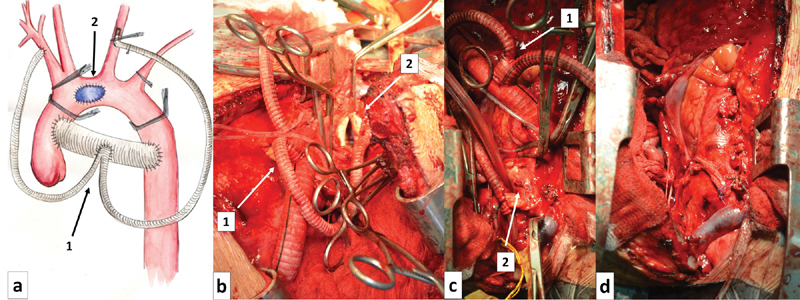
Operation method scheme and intraoperative pictures: ( **a** ) operation schematic, ( **b** ) aortic lumen with aneurysm cavity, ( **c** ) aortic wall restoration, ( **d** ) restoring of the blood flow and the temporary shunt deleting. 1, temporary shunt; 2, aortic arch restoration.

The brachiocephalic trunk was unable to mobilize because it was intimately fused with the anterior wall of the false aneurysm. Therefore, the right subclavian artery was controlled. A temporary bypass (TB) shunt of 20 mm between the ascending and descending aorta was created. In addition, from this bypass an anastomosis with a bifurcation prosthesis for temporary blood supply to the brachiocephalic trunk and left common carotid artery was formed. The first branch of the bifurcated bypass was anastomosed to the right subclavian artery, and the second connected through cannulation to the left carotid artery. The bloodstream was allowed to run through all temporary shunts. The ascending aorta was clamped distal to the shunt, and the descending aorta was clamped proximal to the shunts. Single clamps were placed on the brachiocephalic trunk, left carotid, and left subclavian arteries. Then, a longitudinal aortotomy was made on the front wall of the aortic arch. On the back, the aortic wall was detected, with the defect (with smooth edges, 35 × 20 mm) leading into the cavity of the giant pseudoaneurysm, which was partially filled with old thrombotic material. The posterior aortic wall defect was closed with a Dacron patch. The anterior aortic wall was restored by closing the incision in the aortic wall, with Teflon felt reinforcement. Blood flow was sequentially restored in the aorta and its branches ( Fig. 3 ).

During the entire operation, blood pressure on the right brachial and femoral artery did not flow below 85 and 90 mm Hg. The duration of operation was 480 minutes. The duration of anesthesia was 680 minutes.

Total blood loss was 1,500 mL, with approximately 700 mL from aneurysm cavity. There were no complications after surgery.

On the first day after operation, a right-sided pneumothorax was diagnosed, which was treated with active drainage. On the second day, the patient was extubated.

An additional drainage to the left pleural cavity was implanted on the fifth day due to persistent left-sided limited pneumothorax. The patient was discharged in good condition on the 19th day after the operation.

**Fig. 4:**
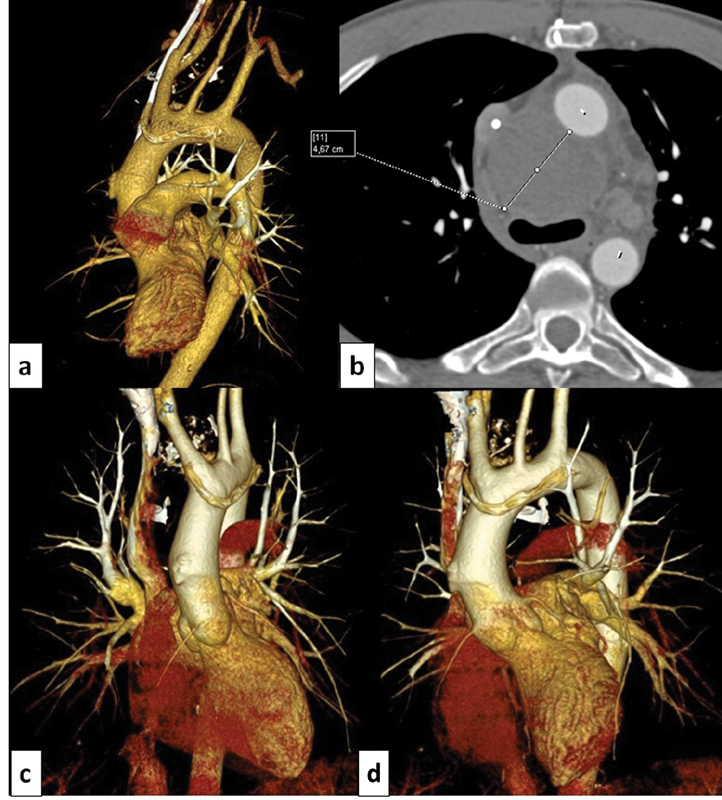
Postoperative CT of the operation zone 15 days after surgery ( **a** , **b** ). Aortic arch aneurysm is isolated from the bloodstream; 8-month follow-up ( **c** , **d** ).

CT scan at 8 months has shown a persistently closed defect ( Fig. 4 ). The size of aneurysm became two times less during 8 months of observation.

## Discussion

Currently, there are two treatment methods for surgery of aortic arch aneurysms. Most often, an aneurysm resection is made with an aortic prosthesis performed using artificial circulation and circulatory arrest with antegrade cerebral perfusion. The second method is a hybrid operation on the aortic arch with complete debranching and implantation of stent graft modules. 2 3 The method of treatment applied by our team for this giant false aneurysm of the aortic arch made it possible to perform this operation in a department without cardiac surgical or endovascular equipment and teams. The use of a TB between the ascending and descending aorta accomplishes an adequate unloading of the left chambers of the heart during the thoracic aorta clamping and provides enough blood flow and pressure to the lower extremities, abdominal aorta, and visceral arteries. Temporary debranching of the brachiocephalic arteries maintains adequate perfusion of the brain.
